# Genetic diversity and population structure of *Brachymystax lenok tsinlingensis* using mitochondrial DNA sequences

**DOI:** 10.1080/23802359.2017.1347897

**Published:** 2017-07-11

**Authors:** Ping Li, Feng Wang, Sien Wen, Hongbao Shen, Xiaoyan Du

**Affiliations:** a Shaanxi Fisheries Institute, Xi’an, China;; b Yellow River Fisheries Research Institute, Chinese Academy of Fishery Sciences, Xi’an, China;; c Jilin Fisheries Research Institute, Changchun, China

**Keywords:** *Brachymystax lenok tsinlingensis*, *cytochrome b*, D-loop, genetic variation

## Abstract

*Brachymystax lenok tsinlingensis* is an endangered freshwater fish which is endemic to part of China. To investigate its genetic diversity and population structure, mitochondrial *cytochrome b* gene and D-loop control region were used to analyze samples from five different locations. Fifteen haplotypes were identified; however, no shared haplotypes were observed among different streams. The analysis of molecular variance (AMOVA) results indicated that 77.38% of total variation was attributed to differentiation between populations, whereas 22.62% from variation within populations. The high genetic differentiation among the populations would provide useful information for building natural reserves and artificially releasing cultured juveniles in the future.

*Brachymystax lenok tsinlingensis* is endemic to several streams of the middle part of the Qinling Mountains, especially in the Heihe, Shitouhe, Xushui and Taibaihe Rivers (Froese and Pauly 2012). Owing to the deterioration of natural habitat and over-exploitation, the wild stocks of *B. lenok tsinlingensis* have experienced dramatic population declines. In 1998, *B. lenok tsinlingensis* was listed as ‘vulnerable’ in China Red Data Book of Endangered Animals and classified as a second-class state protected wild aquatic animals in China (Sung et al. 1998). Nowadays, people are trying to build natural reserves and artificially reproduce and release juveniles to protect natural resources. Moreover, the classification between *B. lenok tsinlingensis* and the *B. lenok* in the north-eastern part of China has been controversial for a long time due to their similar morphological characteristics. Here in this study, mitochondrial DNA (*cytochrome b* gene and D-loop) will be applied to analyze population genetic diversity and genetic relationships of samples from different locations due to its rapid evolution rates and maternal inheritance (Lee et al. 1995; Apostolidis et al. 1997). The results would be useful for the management of germplasm resource and conservation of wild *B. lenok tsinlingensis*.

Specimens of *B. lenok tsinlingensis* were collected from Yanji (S2, *n* = 9) and Baoji (S1, S3, S4 and S5, n = 4*9 = 36) (Figure 1). All the work was conducted with the formal approval of the animal care committees. Fish sampling was done using fishing nets. Total genomic DNA was extracted from approximately 100 mg pectoral fin following a modified phenol-chloroform procedure (Wasko et al. 2003). Mitochondrial genome sequences were downloaded from GenBank with accession number of JQ686731.1 and were used to design primers for *cytochrome b* gene and D-loop by Primer Premier 5 (Lalitha 2000). The partial sequences of mitochondrial *cytochrome b* gene were amplified with primers 5′ CAA GGC AGC AAA GTA GGG 3′ and 5′ CTC CGA TCT CCG GAT TAC AAG AC 3′, and partial sequences of mitochondrial D-loop were amplified with primers 5′ AGA GCG CCG GTG TTG TAA TC 3′ and 5′ GCT AGC GGG ACT TTC TAG GGT 3′. Polymerase chain reaction (PCR) was carried out in 50 μL mix containing 1 X PCR reaction Buffer (including Mg), 0.2 mM dNTP, 1 μM forward and reverse primers, 1.25 units of *Taq* DNA polymerase (TaKaRa) and about 500 ng of genomic DNA. All PCRs were performed with an initial denaturation of 94 °C for 3 min, followed by 35 cycles of 94 °C for 30 s, annealing temperature of 60 °C for 30 s, 72 °C for 90 s and the final extension of 10 min at 72 °C. PCR products were sent to Shanghai Sangon Biotech Co. Ltd. (Shanghai, China) for sequencing.

**Figure 1.**
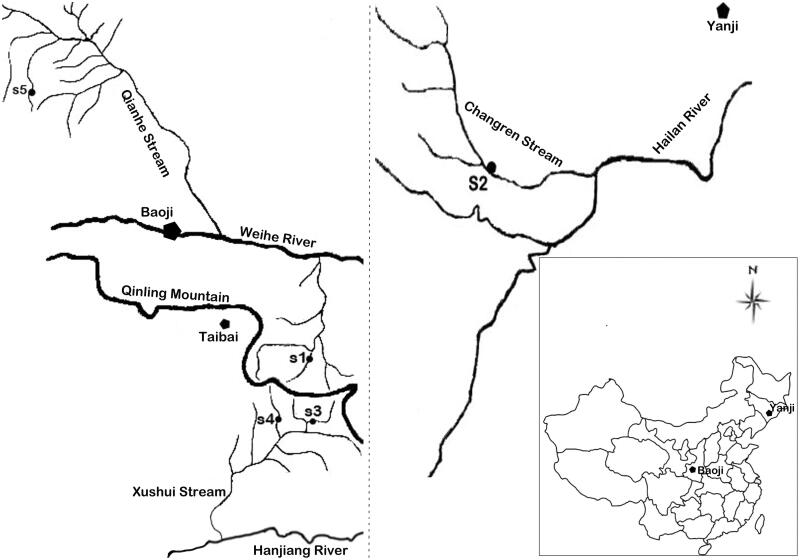
Sampling locations of the five *Brachymystax lenok tsinlingensis* populations. S1 and S5 are from streams of Weihe River (a tributary of Yellow River). S3 and S4 are from Xushui Stream of Hanjiang River (a tributary of Yangtze River). S2 is from Changren Stream of Hailan River in the northeastern part of China.

DNA sequences were aligned and edited by ClustalW (Thomopson et al. 1994). AMAS (Borowiec 2016) was used to concatenate *cytochrome b* sequence and D-loop region into one sequence. Haplotypes (*H*), haplotype diversity (*Hd*) and nucleotide diversity (π) were calculated for within and between different geographic population using DnaSP v5 (Librado and Rozas 2009). MEGA 6.06 (Tamura et al. 2013) was utilized to select Hasegawa–Kishino–Yano (HKY) model and gamma distributed (G) rates among sites for construction of a maximum likelihood (ML) tree. A neighbour joining (NJ) tree based on Kimura 2-parameter model was also generated using MEGA 6.06. Mitochondrial genome sequences of two isolates of *Brachymystax tumensis* were downloaded from the GenBank with the accession number of NC_024674.1 and KJ730525.1 and were used as outgroups in the construction of the phylogenetic trees. Gaps were removed by complete deletion and the bootstrapping with 1000 replications was conducted to evaluate the phylogenetic trees. Arlequin version 3.5.2.2 (Excoffier et al. 2005) was used to estimate genetic differentiation of *B. lenok tsinlingensis* populations by the analysis of molecular variance (AMOVA; (Excoffier et al. 1992)), in which S3 and S4 samples were combined as one population.

Alignment of 45 concatenated sequences resulted in 2049 sites, of which 64 were variable and parsimony informative. Fifteen haplotypes were identified, but no shared haplotypes were observed among different streams. The haplotype sequences of *cytochrome b* and D-loop were uploaded to GenBank separately with the accession number of MF373745 – MF373757. S2 individuals were the most diverse with *Hd* of 0.972 ± 0.064 and π of 0.00198, followed by *Hd* of 0.917 ± 0.073 and π of 0.00073 in S5 specimens. S1 individuals shared one haplotype, and S3 and S4 samples shared the same two haplotypes, corresponding to the same sampling streams. The ML tree and NJ tree suggested the same phylogenetic relationships with four clades (Figure S1), namely S1, S2, S3-S4 and S5, which referred to four geographical locations (Figure 1). Based on the results, the *B. lenok* from Jilin province of China differs greatly from *B. lenok tsinlingensis*. The AMOVA results of *B. lenok tsinlingensis* populations indicated that 77.38% of molecular variance was attributed to differentiation between populations, whereas 22.62% from variation within populations. The differentiation of haplotypes in different *B. lenok tsinlingensis* populations was consistent with the AMOVA results and phylogenetic analyses, indicating genetic variation exists among different streams. Moreover, the genetic diversity is low in certain locations (S1, S3 and S4) according to this study. To further investigate genetic diversity and genetic distance among populations in different streams, more samples from more streams need to be collected. In conclusion, the findings in this study would be useful for building natural reserves and artificially releasing cultured *B. lenok tsinlingensis* juveniles in the future.

## Supplementary Material

TMDN_A_1347897_Supplementary_Information.zip
