# Supplementary figures and images for: Genetic diversity and population structure of Brachymystax lenok tsinlingensis using mitochondrial DNA sequences
Source: Mitochondrial DNA B Resour. 2017 Jul 11;2(2):408–10. doi: 10.1080/23802359.2017.1347897 (PMC7800879; doi:10.1080/23802359.2017.1347897)

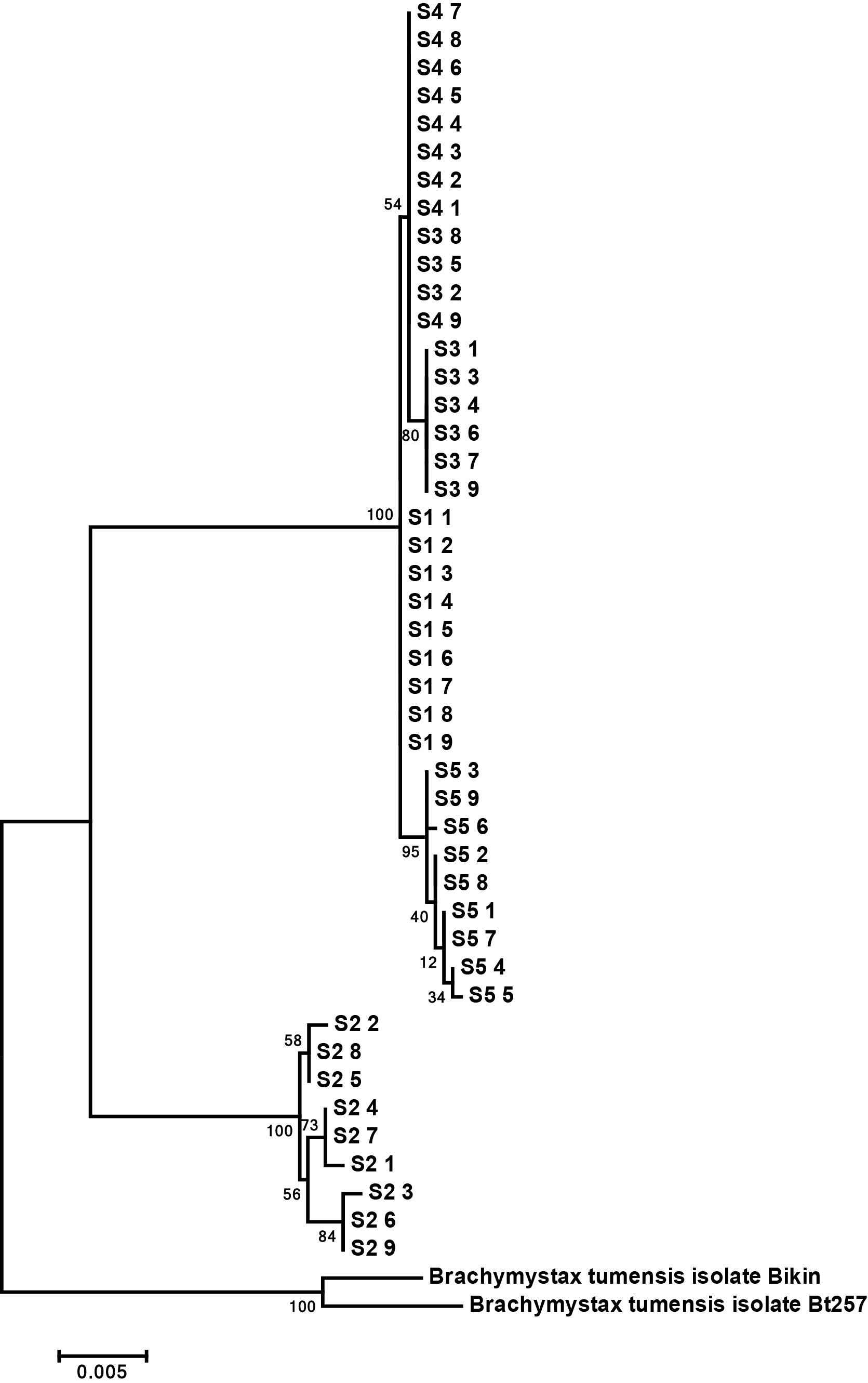

Supplement: TMDN_A_1347897_Supplementary_Information.zip [file TMDN_A_1347897_SM4438.zip › TMDN_A_1347897_Supplementary_Information.jpg]
